# Synergistic Effects of Sulfated Polysaccharides from Mexican Seaweeds against Measles Virus

**DOI:** 10.1155/2016/8502123

**Published:** 2016-06-22

**Authors:** Karla Morán-Santibañez, Lucia Elizabeth Cruz-Suárez, Denis Ricque-Marie, Daniel Robledo, Yolanda Freile-Pelegrín, Mario A. Peña-Hernández, Cristina Rodríguez-Padilla, Laura M. Trejo-Avila

**Affiliations:** ^1^Laboratorio de Inmunología y Virología, Facultad de Ciencias Biológicas, Universidad Autónoma de Nuevo León, Ciudad Universitaria, CP 66455, San Nicolás de los Garza, NL, Mexico; ^2^Programa Maricultura, Facultad de Ciencias Biológicas, Universidad Autónoma de Nuevo León, Ciudad Universitaria, CP 66455, San Nicolás de los Garza, NL, Mexico; ^3^Cinvestav Unidad Mérida, Km 6 Carretera Antigua a Progreso, Cordemex, AP 73, 97310 Mérida, YUC, Mexico

## Abstract

Sulfated polysaccharides (SPs) extracted from five seaweed samples collected or cultivated in Mexico (*Macrocystis pyrifera*,* Eisenia arborea*,* Pelvetia compressa*,* Ulva intestinalis*, and* Solieria filiformis*) were tested in this study in order to evaluate their effect on measles virus* in vitro*. All polysaccharides showed antiviral activity (as measured by the reduction of syncytia formation) and low cytotoxicity (MTT assay) at inhibitory concentrations. SPs from* Eisenia arborea* and* Solieria filiformis* showed the highest antiviral activities (confirmed by qPCR) and were selected to determine their combined effect. Their synergistic effect was observed at low concentrations (0.0274 *μ*g/mL and 0.011 *μ*g/mL of* E. arborea* and* S. filiformis* SPs, resp.), which exhibited by far a higher inhibitory effect (96% syncytia reduction) in comparison to the individual SP effects (50% inhibition with 0.275 *μ*g/mL and 0.985 *μ*g/mL of* E. arborea* and* S. filiformis*, resp.). Time of addition experiments and viral penetration assays suggest that best activities of these SPs occur at different stages of infection. The synergistic effect would allow reducing the treatment dose and toxicity and minimizing or delaying the induction of antiviral resistance; sulfated polysaccharides of the tested seaweed species thus appear as promising candidates for the development of natural antiviral agents.

## 1. Introduction

Latin America has an important and diverse group of seaweed species [1]. Recent data on seaweed management in this region have described the main harvest and aquaculture taking place in Argentina, Brazil, Chile, Peru, and Mexico [2]. One of the goals of seaweeds exploitation is to diversify their application by screening their diverse bioactive compounds, which remain unexplored in nutraceutical and pharmaceutical areas [3]. Different chemical compounds have been isolated from algae, including polysaccharides, which have been subjected to a variety of studies due to their extensive bioactivities and applications [4].

An increasing number of biological activities of seaweed polysaccharides have been reported in the last decades, where sulfated polysaccharides (SPs) are among the most studied compounds [5]. SPs include a complex group of macromolecules with numerous activities such as antioxidant [6, 7], antitumor [8, 9], anticoagulant [6], anti-inflammatory [6, 10], and antiviral [11, 12].

Antiviral activity of SPs was first reported in 1958 [13] and over the years a substantial research has been focused on this field. SP can be obtained from each of the three main classes of seaweed: fucoidans and alginates from brown algal species, agaroids and carrageenans from red macroalgae, and ulvans from green seaweeds [14].

Fucoidans have shown a potent antiviral activity against numerous enveloped viruses including herpes simplex virus type 1 (HSV-1) [15], human immunodeficiency virus [16], influenza A virus [17], and different kind of paramyxoviruses such as Newcastle disease virus (NDV) and canine distemper virus (CDV) [18, 19].* In vitro* and* in vivo* antiretroviral effects of alginates preventing syncytium formation and reducing the P24 core antigen level have been demonstrated [20]. Antiviral activity of carrageenans has been demonstrated* in vitro* against human papillomavirus (HPV), acting mainly on the inhibition of HPV virions binding to cells, and also* in vivo* by preventing infection by different HPV genotypes [21, 22]. Recently, antiviral activity against NDV of ulvan from* Ulva clathrata* cultivated in Mexico has been reported [23].

Nowadays, combining multiple drugs is a primary approach for improving antiviral effects within the antiviral drug therapy field. The advantages of multidrugs combination are the reduction of individual drugs doses, a decrease in the side effects of antiviral agents, and the prevention of drug-resistant viruses emergence. Drug combination theories provide an ideal tool for this purpose to understand the benefits of multidrugs combinations therapy [24].

Measles virus (MeV) belongs to the Paramyxoviridae family of Mononegavirales, is a nonsegmented negative-strand RNA virus, and causes a highly contagious disease [25]. Although preventable by vaccination, measles still remains one of the causes of death among young children worldwide [26]. Many new antiviral drugs have been licensed in recent years, most of which are used for the treatment of HIV infections [27]. The investigation of natural antivirals isolated from marine sources is an interesting approach in the development of new antiviral agents. In the present study, we tested the antiviral activity of SPs isolated from five Mexican seaweeds against MeV. The aim of this research was to develop new candidates of antiviral drugs that could help to control viral infection diseases.

## 2. Materials and Methods

### 2.1. Antiviral Agents

#### 2.1.1. Collection of Seaweed

Five species of macroalgae were collected from the Mexican coasts and tested for this study: three brown seaweeds from Baja California (*Macrocystis pyrifera*,* Eisenia arborea*, and* Pelvetia compressa*), one green seaweed from Southern Baja California (*Ulva intestinalis*), and one red seaweed from Yucatan (*Solieria filiformis*).

*Macrocystis pyrifera *(Linnaeus) C. Agardh was collected in Bahía de Ensenada (Manto Jantay) in front of the Salsipuedes beach (31.983–116.815), in January 2013.* Eisenia arborea* J. E. Areschoug and* Pelvetia compressa* (J. Agardh) De Toni were collected in the Escalera Zone, North of Punta China (31.520–116.650) in December 2014-January 2015. The green alga* Ulva intestinalis* (Linnaeus) was collected from the water drainage channel of the Gran Mar shrimp farm, on the Baja California West coast (24.434–111.584) in August 2014.

*Solieria filiformis* (Kützing) P. W. Gabrielson, a red seaweed considered as a potential source of *ι*-carrageenan [28], was obtained from an aquaculture facility at the Telchac Marine station-CINVESTAV, Yucatan (Mexico), where it is periodically cultivated in bimonthly cycles in semiopen tanks as part of an Integrated Multitrophic aquaculture system. The sample used came from a batch cultured from April to May 2014.

Once harvested, the brown and green algae samples were washed in seawater to eliminate sand, shells, and epibionts and dried under shade, while the cultivated red algae was washed with fresh water and dried in an oven at 60°C. Prior to extraction, the samples were cut into small 2-3 cm pieces and ground to pass through a 0.5 mm sieve (Turbomolino Pulvex 200 mill).

#### 2.1.2. Extraction and Purification of Sulfated Polysaccharides

Polysaccharides extraction was performed after extraction of polyphenols [29]. Briefly 10 g of alga powder was washed with distillated water and dried at room temperature overnight. The washed powder was extracted with 200 mL 50% v/v ethanol and sonicated for 30 min at room temperature, followed with an extraction period in a bath shaker at 70°C during 2 hours. The samples were centrifuged for 15 min (2500 rpm). The pellet was used for the polysaccharide extraction according to the procedure described by Tako et al. 2000 and Ale et al. 2012 [30, 31]. Briefly, 200 mL of 0.1 M HCl was added to the algae pellet and heated for 1 hour at boiling temperature and centrifuged at 3500 rpm for 10 minutes. The supernatant was recovered and absolute ethanol was added (4 : 1) for polysaccharides precipitation. Once precipitated, the polysaccharides were separated from the aqueous medium by centrifugation at 3500 rpm for 10 minutes; the supernatant was discarded and the pellet was washed three times with 96% ethanol to remove residual pigments and finally resuspended in a minimum amount of distilled water for a 72-hour dialysis with stirring. The dialyzed product was precipitated with absolute ethanol (4 : 1). Polysaccharide extracts were lyophilized and weighed to calculate their yield.

#### 2.1.3. Characterization of Selected Polysaccharide Extracts

*(1) FT-IR Spectra Analysis*. IR spectra of aqueous extracted polysaccharides from* Solieria filiformis* and* Eisenia arborea* were obtained using diffuse reflectance infrared Fourier transform spectroscopy (DRIFTS). Scans were performed at room temperature in the infrared region between 4000 and 400 cm^−1^ on a Thermo Nicolet Nexus 670 FT-IR spectrometer. The infrared spectra of commercial available carrageenan (*ι*-carrageenan C1138, *κ*-carrageenan C1013), fucoidan from* Fucus vesiculosus* (F5631), alginic acid (A7003) from Sigma-Aldrich (St. Louis, MO, USA), and *λ*-carrageenan from Celtic Colloids Inc. (B. Blakemore) were included for comparison.

*(2) NMR Spectra Analysis*. ^13^C-NMR spectra were acquired on a Varian 600 spectrometer. The extracts were exchanged twice with 99.8% deuterium oxide (D2O) with intermediate lyophilization and dissolved at 10 mg mL^−1^ in D2O. Sodium [3-trimethylsilyl 2,2′,3,3′-2-H4] propionate (TSP-d4) was used as an internal reference to 0.00 ppm.

*(3) Carbohydrate Determination*. For determination of total sugars in the samples acid hydrolysis of the extracts was performed. A solution with 25 mg of polysaccharide extract in 100 mL of 1 M H_2_SO_4_ was prepared and boiled for 3 hours; subsequently, an aliquot of 1 mL of each extract was taken. Anthrone reagent (5 mL) was added to the aliquot, placed in a water bath for 12 minutes, and cooled down at room temperature. Absorbance was read at 630 nm. Quantification was performed against a calibration curve of a stock solution of fucose.

*(4) Sulfate Content Determination*. The analysis was performed using the turbidimetric method of Jackson and McCandless, 1978 [32]. Briefly, this quantification of sulfates was determined by measuring turbidity as barium sulfate when adding 1.2 mL of TCA 8% and 0.6 mL of 0.01% reaction reagent (agarose/barium chloride) to the sample; reaction was homogenized by stirring for 35 minutes. The turbidity was determined at 500 nm in a Shimadzu UV-Vis spectrophotometer 1601. The calibration curve was performed with potassium sulfate (K_2_SO_4_) with a concentration of 0 to 100 *μ*g of SO_4_^−2^/mL. SP extracts of* Solieria filiformis* and* Eisenia arborea* were weighed (7 mg), and 1 mL of 1 N HCl was added and heated at 105°C for 12 hours in a thermoblock (Lab line). A dilution was performed with 10 mL of deionized water; samples were then filtered using a microfilter with Whatman paper of 1.2 *μ*m and an aliquot of 1.1 mL of the samples was taken for quantification. Analysis was performed by triplicate.

### 2.2. Cells and Virus

Vero cells were grown at 37°C in a 5% CO_2_ atmosphere in Dulbecco's Modified Eagle Medium Nutrient Mixture F-12 (DMEM/F12, Gibco Invitrogen, USA) supplemented with 5% fetal bovine serum (FBS, Gibco Invitrogen, USA) and 1% antibiotic (Gibco Invitrogen, USA).

Measles virus (Edmonston strain) was purchased from ATCC (ATCC® VR-24*™*). Virus was propagated on Vero cells and viral titers were determined by cytopathogenic effect and expressed as 50% tissue culture infectious dose (TCID50)/mL. Aliquots of viral stock were stored at −80°C until use.

### 2.3. Cytotoxicity Assays

The effect of SPs on cell viability of Vero cells was determined by MTT assay. The cells were cultured in 96-well plates at a density of 1.5 × 10^4^ cells/well at 37°C in an atmosphere of CO_2_. After 1 day of incubation, increasing concentrations of SPs diluted in DMEM were added; after 48 h of incubation the media were replaced with 22 *μ*L of 2.5 mg/mL MTT dissolved in phosphate-buffered saline (PBS). After 1 h 30 min 150 *μ*L of DMSO was added and incubated at room temperature for 15 min. The optical density (OD450 nm) was measured using a microplate reader (Multiskan FC, Thermo, USA). Cell viability was expressed by percentage as the mean value of three independent experiments considering control cells absorbance as 100% viable. CC_50_ was the concentration of the test substances that inhibited the Vero cells growth by 50% compared with the growth of the untreated cells.

### 2.4. Syncytia Reduction Assays

The antiviral activity of the SPs was evaluated by syncytia reduction assays. Vero cells seeded in 12-well plates were treated with different concentrations of SPs (0.01–5 *μ*g/mL) and infected with MeV (1 × 10^3.5^ TCID50 of Edmonston strain) at the same time. After virus adsorption for 1 h at 37°C the medium was removed and monolayers were washed with PBS, after which the corresponding concentrations of SPs were added again. Each concentration was tested using three culture wells per PS concentration per experiment; the experiments were performed by triplicate. After incubation of 48 or 72 h at 37°C in a 5% CO_2_ incubator monolayers were fixed with methanol : acetone (1 : 1) and stained with 1% crystal violet. Syncytia were counted and the result was expressed as a percentage of the number of syncytia observed in viral control monolayers (untreated cultures); IC_50_ was determined from dose-response curves. The selectivity index (SI) values were calculated as CC_50_/IC_50_. SPs showing the best SI were selected for the subsequent experiments.

### 2.5. Quantitative Real-Time PCR

Total RNA was isolated from treated Vero cells using RNAzol® RT (MRC Inc., USA). Reverse transcription was performed using the High Capacity cDNA Reverse Transcription Kit (Applied Biosystems, USA) and the viral genome was amplified with specific primers (MeVF: 5′ GAGGGTCAAACAGAGTCGAG 3′, MeVR: 5′ CGGTTGGAAGATGGGCAG 3′) that amplified a 95 nt fragment. The real-time PCR was carried out using SensiFAST*™* SYBR® No-ROX Kit (BIOLINE, USA) and the Chromo4*™* Real-Time PCR Detector (Bio-Rad, USA) with the following procedures: 95°C for 2 min, followed by 50 cycles of 95°C for 2 s, 60°C for 10 s, and 72°C for 20 s. The number of viral copies was calculated by using a standard curve. Serial 10-fold dilutions of a synthetic oligonucleotide encompassing the target measles gene were used to establish the standard curves.

### 2.6. Evaluation of SPs Synergy

Potential synergistic effects of selected SPs on MeV infection were evaluated using syncytia reduction assays. Each combination was tested on its corresponding IC_75_, IC_50_, and IC_25_ values. The synergistic effect of SPs was calculated by using a combination index (CI) described previously by Chou [33] and CompuSyn software. CI was calculated from the data as a measure of the interaction among drugs. CI values lower than 0.9 indicate synergy, CI values from 0.9 to 1.1 indicate an additive effect, and CI values higher than 1.1 indicate antagonism. Combinations with synergistic antiviral effect were selected and qPCR assays were performed in order to confirm the inhibitory effect as described above.

### 2.7. Time of Addition Assay

Vero cell monolayers were infected with MeV. SPs were added at a concentration of 5 *μ*g/mL at different times of infection: 60 min before infection and 0, 15, 30, 60, and 120 min after infection. Thereafter, for each treatment, cells were incubated with SP for 1 h and then washed three times with PBS. Monolayers were fixed with methanol : acetone after incubation for 48 or 72 h at 37°C and 5% CO_2_ and stained with 1% crystal violet; syncytia were counted subsequently.

### 2.8. Viral Penetration Assay

Virus penetration into Vero cells was evaluated according to the method reported by Huang and Wagner [34] with some modifications [18]. Vero cell monolayers precooled at 4°C for 3 h were infected with MeV at 4°C for 1 h in the absence of SP. After washing three times with ice-cold PBS, different concentrations of SP were added to the monolayers, and the temperature was shifted to 37°C. After 1 h of incubation at 37°C, the cells were treated with 40 mM citrate buffer (pH 3.0) to inactivate unpenetrated viruses. Buffer was replaced by culture medium and the cells were incubated for 48 or 72 h at 37°C and 5% CO_2_ and stained with 1% crystal violet; syncytia were counted subsequently.

### 2.9. Statistical Analysis

The variables (tested by triplicate in each experiment that were in turn repeated at least three times) were submitted to a one-way analysis of variance followed by Dunnett's test (SPSS software, *α* = 0.05). CC_50_ and IC_50_ values were determined by probit regression analysis.

## 3. Results

### 3.1. Cytotoxicity and Antiviral Activity of SPs

The MTT assay indicated no cytotoxicity for any of the SPs at concentrations from 0.1 to 1500 *μ*g/mL up to 2 days (data not shown).

Antiviral activity of SPs against MeV was evaluated by syncytia reduction inhibition assays at concentrations of 0.01, 0.1, 1, and 5 *μ*g/mL of each compound (data not shown). All tested compounds showed significant antiviral activity, but only compounds with the best SI values were selected for the subsequent experiments. As shown in Table 1, SPs of* Eisenia arborea* and* Solieria filiformis* exhibited antiviral activity at the lowest concentrations (IC_50_ 0.275 *μ*g/mL and 0.985 *μ*g/mL, resp.) without cytotoxic effect at concentrations of 0.1 to 1500 *μ*g/mL. Therefore, SPs of* Eisenia arborea* and* Solieria filiformis* were selected based on their SI and antiviral activity for the combination experiments.

**Figure 1:**
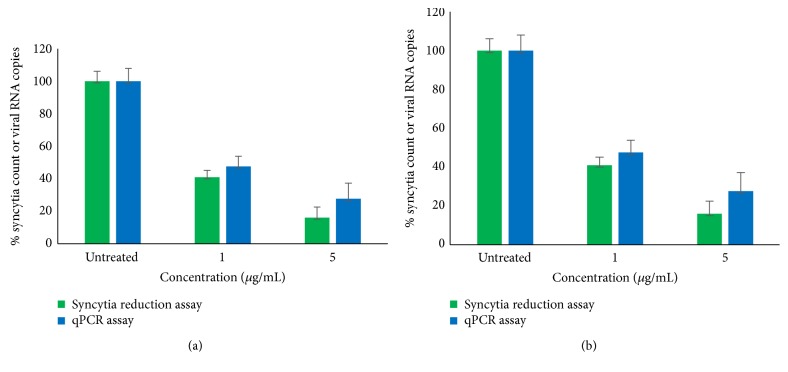
Confirmation of antiviral activity of* Eisenia arborea* (a) and* Solieria filiformis* (b) SPs at their best inhibitory concentrations by syncytia reduction and qPCR assays. Syncytia count and viral RNA copies number are given in % of the untreated control values.

Antiviral effect of selected SPs was confirmed by qPCR assays, as shown in Figure 1. Inhibitory effect of* Eisenia arborea* and* Solieria filiformis* SP was tested at the best inhibitory concentrations (1 *μ*g/mL and 5 *μ*g/mL for both SPs). Results of qPCR assays were consistent with the results observed by syncytia reduction inhibition assays.

### 3.2. Characterization of SPs

**Figure 2:**
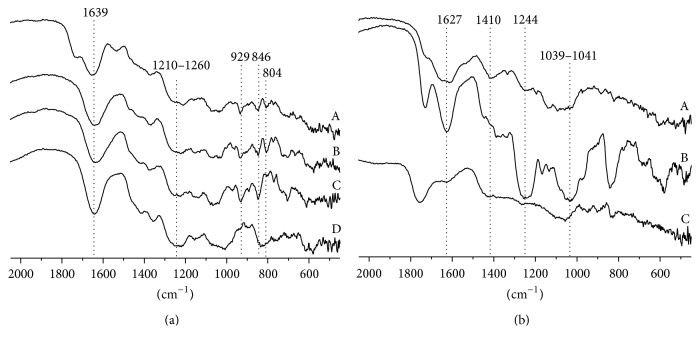
(a) Infrared spectra of (A)* Solieria filiformis *aqueous extract, (B) *ι*-carrageenan, (C) *κ*-carrageenan, and (D) *λ*-carrageenan. (b) Infrared spectra of (A)* Eisenia arborea* aqueous extract, (B) fucoidan, and (C) alginic acid.

Infrared spectroscopy has been used for the qualitative characterization of carrageenans and has proven to be a valuable tool for the characterization of sulfated oligosaccharides [35]. FT-IR and NMR spectra analyses of selected SPs extracts were performed. The FT-IR spectrum of* Solieria filiformis* extract (Figure 2(a)) indicates the presence of a typical *ι*-carrageenan type. Characteristics signal bands are indicated: 3,6 anhydrogalactose-2-sulfate (804 cm^−1^) characteristic of *ι*-carrageenan; galactose-4-sulfate (846 cm^−1^) signal present in *κ*- and *ι*-carrageenan. The signal between 1210 and 1260 cm^−1^ is common to all types of compounds containing sulfate.

**Figure 3:**
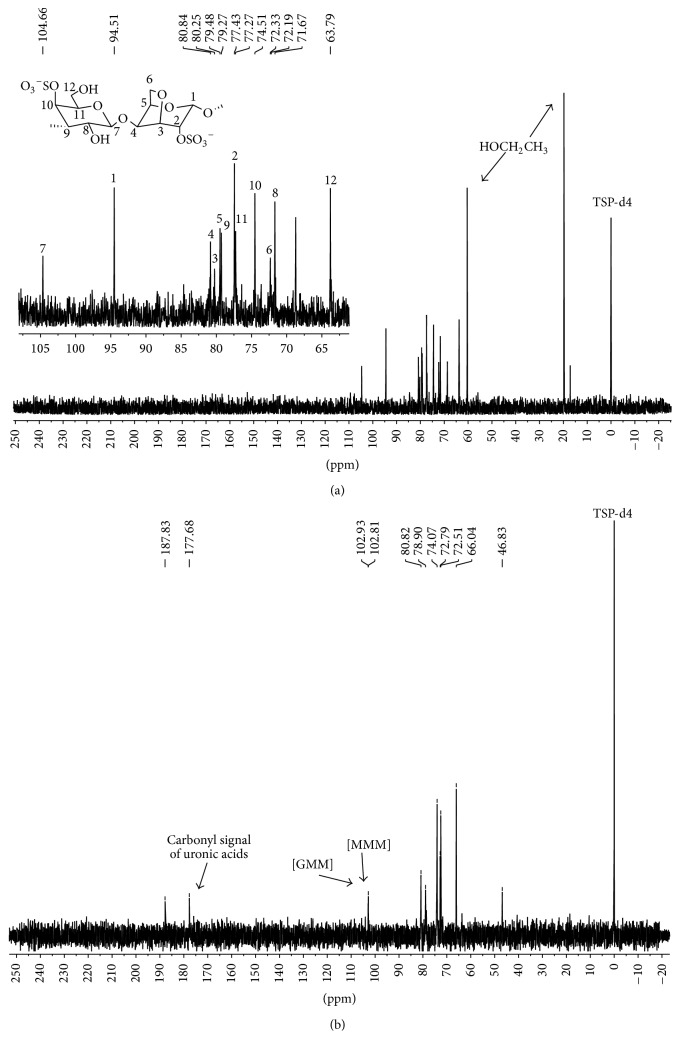
(a) Spectrum and expansion ^13^C-NMR of the aqueous extract of* S. filiformis*. (b) ^13^C-NMR spectrum of the aqueous extract of* E. arborea*.

^13^C-NMR spectroscopy has been highly recommended for distinguishing the polysaccharides of the agar and carrageenan group [36]. Spectrum and expansion ^13^C-NMR of the* S. filiformis* extract (Figure 3(a)) showed signals at 20 and 60 ppm typical of residual ethanol. Carbohydrates signals (63.79–104.66 ppm) observed, particularly two upfield-shifted signals (94.51 and 104.66 ppm), suggest that the molecule has two anomeric carbons. Overall its spectrum showed the presence of the *ι*-carrageenan. The next assignment is the mapping of the carbon signals of the molecule. Carbons of 2-sulfate-3,6-anhydrogalactose are 94.51 (C1), 77.44 (C2), 80.25 (C3), 80.84 (C4), 79.49 (C5), and 72.33 (C6) ppm [37]. Carbons of 4-sulfate-galactose are 104.66 (C1), 71.68 (C2), 79.27 (C3), 74.51 (C4), 77.27 (C5), and 63.79 (C6) ppm [37]. Sulfate content of* S. filiformis* showed 21.14% (±0.056) of total sulfate and total polysaccharide determination resulted in 91% of polysaccharide.

The FT-IR spectrum of* Eisenia arborea* extract (Figure 2(b)) indicates the presence of a mixture of fucoidan and alginic acid. Characteristics signal bands are indicated: carboxylate vibrations (1627 and 1410 cm^−1^) can be attributed to uronic acids. Stretching vibrations at 1039–1041 cm^−1^ can be assigned to pyranose ring from guluronic and mannuronic acid residues. The broad band at 1244 cm^−1^ indicates the presence of sulfated ester groups, which are characteristic in fucoidans. ^13^C-NMR spectrum of* E. arborea* extract (Figure 3(b)) showed typical signals of alginate ranging from 66.04 to 177.68 ppm. The signal at 66.04 ppm is characteristic of carbon-2 of guluronic acid (G) [38]. The signals at 72.51, 72.79, 74.07, 78.90, 80.82, 102.81, 102.93, and 177.68 ppm correspond to repeating blocks of mannuronic (M) and guluronic acid [39]. The signals at 102.81 and 102.93 ppm may indicate the presence of two repeating units, one of MMM and another of GMM [39]. Sulfate content of* E. arborea* showed 12.85% (±0.346) of total sulfate.

### 3.3. Combined Antiviral Effect of SPs

The combined effect of SPs of* Eisenia arborea* and* Solieria filiformis* on MeV infections was examined: each SP was tested at different concentrations combining its corresponding IC_25_, IC_50_, and IC_75_ values. E_25_, E_50_, and E_75_ correspond to IC_25_, IC_50_, and IC_75_ values of* Eisenia arborea* SPs and S_25_, S_50_, and S_75_ correspond to the respective values of* Solieria filiformis* SP (Table 2). Syncytia reduction assay results were expressed in relative syncytia percentage according to the number of syncytia in viral control. Best inhibitory effect was observed for E_50_-S_25_ combination.

**Figure 4:**
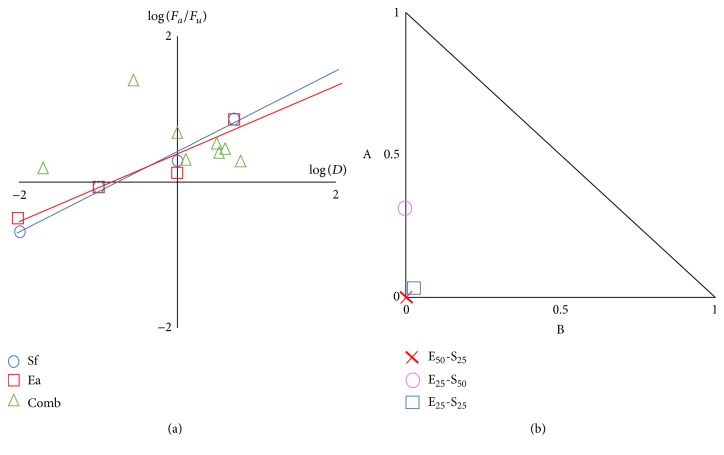
Analysis of* Eisenia arborea* and* Solieria filiformis* combinations. (a) Median-effect plot for combinations of* Eisenia arborea* and* Solieria filiformis* was generated with the CompuSyn software (*F*_*a*_, affected fraction; *F*_*u*_, unaffected fraction; *D*, concentration of SP used; Sf* Solieria filiformis* SP; Ea,* Eisenia arborea* SP; Comb,* Eisenia arborea* and* Solieria filiformis* combinations). (b) Normalized isobologram plots for Sf and Ea at nonconstant combination ratios. For each SP different combinations of various concentrations based on IC_25_, IC_50_ values were tested and combination index (CI) values were determined using the CompuSyn software. CI values, represented by points below the lines, indicate synergy.

The evaluation of drug synergism based on a median-effect equation has been extensively used in the literature. CI values of SPs combinations were calculated as described previously using the CompuSyn software and are given in Table 2. Median-effect and the normalized isobologram generated with the software determined the presence of three synergistic combinations, represented by points below the lines at normalized isobologram (Figure 4).

**Figure 5:**
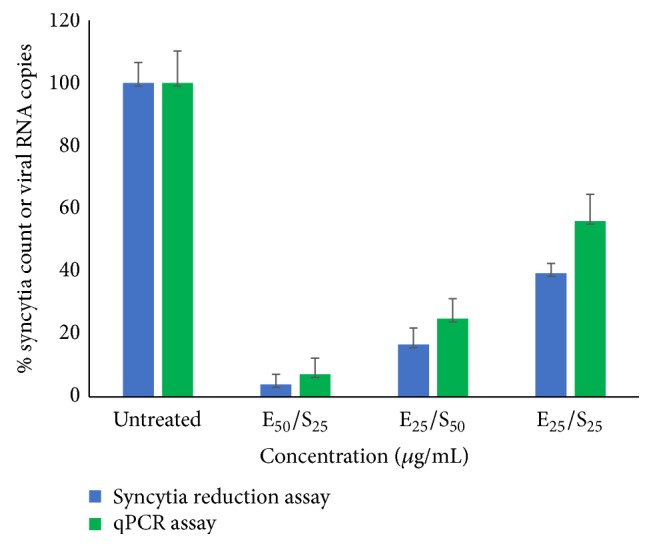
Antiviral activity confirmation by qPCR of the RNA extracted from Vero cells infected with MeV and cultivated in presence of synergistic SPs combinations. E_25_ and E_50_ are the SPs concentrations corresponding to IC_25_ and IC_50_ values of* Eisenia arborea* SPs. S_25_ and S_50_ concentrations correspond to the respective IC values of* Solieria filiformis* SP.

Results showed strong synergistic effects at low concentrations combinations (E_50_-S_25_, E_25_-S_50_, and E_25_-S_25_) and antagonism at high concentrations combinations (E_25_-S_75_, E_50_-S_50_, E_50_-S_75_, E_75_-S_25_, E_70_-S_50_, and E_75_-S_75_). Combinations with synergistic effect were selected and qPCR assays were performed. As shown in Figure 5 the inhibitory effect of the synergistic combinations was confirmed. These data were consistent with results observed by syncytia reduction inhibition assays.

### 3.4. Effect of SPs on Viral Infection at Different Times of Addition

**Figure 6:**
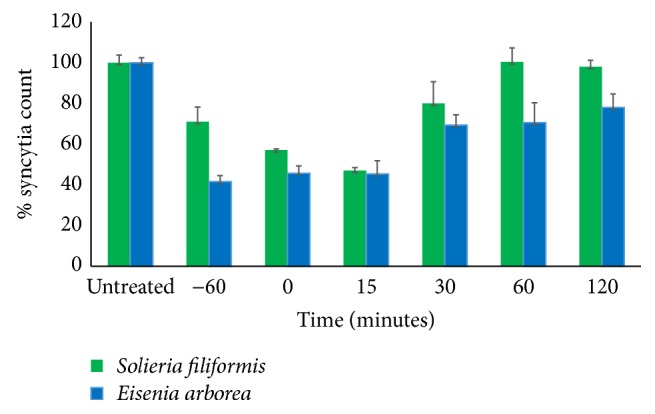
Time of addition experiments. Antiviral activity of SP was tested at different times of infection and analyzed by syncytia inhibition assays. SPs were added at 60 min before infection and 0, 15, 30, 60, and 120 min after infection. The data are expressed as relative syncytia count (%) compared to that of untreated virus-infected control cells, which was defined as 100%. The data shown are the mean ± SD of triplicate experiments.

In order to determine which step of the MeV cycle was targeted by SPs, “time of addition” experiments were performed in Vero cells infected with MeV and exposed to PS at different times of infection. The most efficient inhibition by* S. filiformis* was observed in early phases of infection, 0 and 15 min after infection (Figure 6); syncytia inhibition before infection and 30 min after infection was not significant.* E. arborea* showed the most efficient inhibition 1 hour before infection and 0 and 15 min after infection. At 30, 60, and 120 min after infection, a minimal syncytia inhibition by* E. arborea* was still observed.

### 3.5. Effect of Fucoidan on Viral Penetration into Host Cells

**Figure 7:**
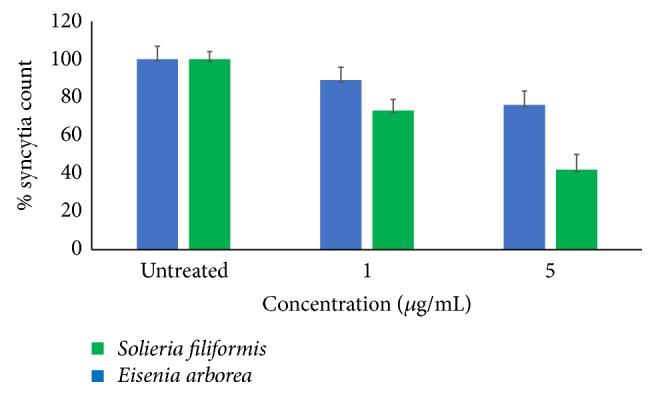
Effect of SPs on viral penetration. Vero cells were infected with MeV at 4°C in the absence of SPs and then shifted to 37°C to permit penetration of the adsorbed virus in the presence of SPs. Antiviral effect of SPs was evaluated using syncytia inhibition assays. The data shown are the mean ± SD of triplicate experiments.

Viral penetration assays were performed to determine whether entry events downstream of virus binding were inhibited by SPs. Vero cells were plated and incubated with MeV at 4°C for 1 h to allow virus binding but prevent viral internalization. Unbound virus was inactivated and SPs (1 *μ*g/mL or 5 *μ*g/mL) were added to the cells and incubated at 37°C.  Figure 7 shows that SP from* S. filiformis* (5 *μ*g/mL) significantly decreased viral infection by 58%, while SPs from* E. arborea* (5 *μ*g/mL) decreased viral infection only by 24%, when compared with the findings in infected cells in the absence of treatment.

## 4. Discussion

Since the first studies by Gerber in 1958 showing the inhibition of mumps and influenza B virus by marine algae polysaccharides, increased efforts and research have been carried out in this field [13]. Previous studies have also demonstrated no cytotoxicity of SPs isolated from certain seaweed species [40]. The absence of cytotoxicity to the host cells is one of the principal challenges in the development of new antivirals.

*Eisenia arborea*, an edible brown alga used in folk medicine in Japan, is the kelp species with the largest and most southerly latitudinal distribution on the North Pacific East Coast [41, 42]. Researches on* Eisenia* biological activities have been focused on the evaluation of their polyphenolic compounds [43]. To our knowledge, the antiviral effects of* Eisenia arborea* extracts have never been tested before. In this study, the extract of* Eisenia arborea* is rich in fucoidans and alginates and also showed the best SI of the five seaweed extracts (Table 1). Previous chemical characterization of Mexican* E. arborea* extracts also reported the presence of alginates, with higher yields than the one reported in this study [44]. Alginates with antiviral effects have been previously tested against HIV, IAV, and HBV, showing a potent antiviral activity [4]. Antiviral activity of fucoidan has been also reported* in vitro* and* in vivo* against many RNA and DNA viruses such as HIV, HSV1-2, dengue virus, and influenza virus [39, 45–47].

*Macrocystis pyrifera* has been harvested since 1956 along the Pacific coast of Baja California and exported to the United States for the production of alginates [48]. SPs extracts of Mexican* Macrocystis pyrifera* showed a significant antiviral effect but were not selected for subsequent assays because of their IC_50_ value (Table 1). Previous studies with crude dialyzed extracts of* Macrocystis pyrifera* have shown antiviral effects against VSV, with the fucoidan being responsible for these results [49].

In this study, antiviral effects of the extract from* Solieria filiformis* display the second lowest IC_50_ among the analyzed extracts.* In vitro* studies have reported antiviral properties of carrageenans against DNA and RNA viruses [21, 50]. Recently it has been shown that carrageenan (Rong Yuan F.F.I. Co., Ltd.) can inhibit influenza virus A/Swine/Shandong/731/2009 H1N1 (SW731) responsible for the influenza pandemic of 2009. Carrageenans can significantly inhibit SW731 replication by interfering with different steps of viral replication, including adsorption, transcription, and expression of the viral proteins; however, they act especially by inhibiting the interactions between the viral receptor (HA) and the target cell [51]. Sulfate content analysis and total polysaccharide determination of* S. filiformis* extract resulted in 21.14% (±0.056) total sulfate and 91% polysaccharide; these data are consistent with previous reports [52]. Degree of sulfation has a major impact on the antiviral activity of polysaccharides, including carrageenans [53].

In relation to the combination therapy approach used in this study, results showed a strong synergistic effect at low concentrations combinations of SPs and antagonism at high concentrations combinations. Our results determined that low concentrations combinations (0.0274 *μ*g/mL and 0.011 *μ*g/mL of* E. arborea* and* S. filiformis*, resp.) exhibited the higher inhibitory effect (96%) in comparison to the individual effect of SP (50% of inhibition with 0.275 *μ*g/mL and 0.985 *μ*g/mL of* E. arborea* and* S. filiformis*, resp.). Synergistic effect observed in this study has been also reported for the sulfated polysaccharides from* Fucus vesiculosus* in combination with AZT against HIV [54]. Furthermore, this effect has been also observed with acyclovir in combination with 3, 19-isopropylideneandrographolide against herpes simplex virus (wild type) and drug-resistant strains. Low concentrations of these compounds were required for a complete inhibition of DNA replication and late protein synthesis of HSV-1 wild type and drug-resistant HSV-1 [55]. The combined effect of nitazoxanide with neuraminidase inhibitors against influenza A viruses tested* in vitro* suggests that regimens that combine neuraminidase inhibitors and nitazoxanide exert synergistic anti-influenza effects [56]. In contrast, antagonistic effects at high concentrations were observed in our study; this antagonism of SPs was previously observed in a combination of ulvan and fucoidan against NDV infection [23]. Particular chemical features of SPs like chain ramifications could explain antagonism effects of SPs. Moreover, carbohydrate to carbohydrate interactions could be responsible to adhesion events; these aggregates have been previously observed in marine sponges [57].

To understand if a synergistic effect was related to different modes of action of the tested SPs, viral penetration and time of addition assays were performed. Results suggested the possibility that SP from* S. filiformis* inhibits postbinding events, because best inhibition effect was observed at 0 and 15 minutes after viral infection (Figure 6). To support this idea, a viral penetration assay was performed (Figure 7), and results show the best antiviral effect after viral adsorption. Our results are in agreement with those observed by Elizondo-Gonzalez et al. [18], who demonstrated the ability of fucoidan from* C. okamuranus* to be responsible for the antiviral activity against Newcastle disease virus, suggesting that fucoidan inhibits viral penetration into host cells, must probably by blocking the F protein.

Similar results were also observed by Bouhlal et al. [58], who suggested that carrageenans can inhibit DENV replication by interfering viral entrance, but they also suggested that SPs could avoid viral adsorption into the cell as a second mode of action. This mode of action could be similar to the mechanism observed with SPs of* E. arborea*. Alginates and fucoidan of* E. arborea* were able to show the best antiviral effect 1 hour before infection and this effect lasted up to 0–15 minutes after infection. Although both SPs, from* S. filiformis* and* E. arborea*, exhibited antiviral activity at 0 and 15 min after infection, only* E. arborea* showed inhibitory effect at 60 min. This result suggests the capability of these SPs to avoid viral adsorption to the cell; these data were confirmed by viral penetration assays where we observed less antiviral activity after viral attachment to the cell. More recent studies have demonstrated that fucoidans exhibit their antiviral activity when the compound is present during the virus adsorption period by blocking the interaction of viruses to the cells [59].

SPs tested in this study exhibit the best antiviral effect at different stages of infection: viral penetration and viral adsorption (*S. filiformis* and* E. arborea*, resp.). Multiple-drug antiviral therapy with two or more drugs that target different proteins or act in different stages of infection may decrease drug resistance and may enhance clinical outcomes by allowing a reduction of individual drug doses, thus decreasing dose-related drug toxicity [60].

## 5. Conclusions

In this study sulfated polysaccharides from Mexican seaweed showed antiviral activity against measles virus. Due to the lack of cytotoxicity at inhibitory concentrations, as indicated by the selectivity index, potential application can be found for these SPs.* Eisenia arborea* and* Solieria filiformis* extracts showed the higher antiviral activity and were selected to determine their combined effect. Synergistic effect was observed at the lowest concentrations tested for each SP of these species. Results suggest that SPs combined in this study are acting at different level of first stages in viral infection. Synergistic therapeutic effect allows dose and toxicity reduction and would minimize or delay the induction of antiviral resistance. Sulfated polysaccharides of Mexican seaweed are potential candidates for the development of new antiviral drugs that can help to control viral infection diseases.

## Figures and Tables

**Table 1:** Cytotoxic effect, antiviral activity, and selectivity index of SPs.

Algae^a^	CC_50_ (*μ*g/mL)^b^	IC_50_ (*μ*g/mL)^c^	SI^d^
*Macrocystis pyrifera*	>1500	1.00	>1500
*Eisenia arborea*	>1500	0.275	>5454.54
*Pelvetia compressa*	>1500	1.00	>1500
*Ulva intestinalis*	>1500	3.6	>416.7
*Solieria filiformis*	>1500	0.985	>1522.84

^a^Algal sulfated polysaccharide extract. ^b^Concentration of test compound (*μ*g/mL) that reduced Vero cell viability by 50%. ^c^Concentration of a test compound that reduced the number of MeV syncytia in Vero cells by 50%. ^d^Selectivity index value.

**Table 2:** Synergistic effects of SPs on MeV infection.

Compounds combination	Compound concentration (*μ*g/mL)		% relative syncytia formation in presence of the different SPs combinations	SD	CI	Description
	*Eisenia arborea*	*Solieria filiformis*				
IC_75_-IC_75_	2.98	3.027	34.5	4.4	10.59	Antagonism
IC_75_-IC_50_	2.98	0.985	26.4	5.6	3.08	Antagonism
IC_75_-IC_25_	2.98	0.011	33	7.3	1.47	Antagonism
IC_50_-IC_75_	0.275	3.027	28.4	4.1	3.71	Antagonism
IC_50_-IC_50_	0.275	0.985	32	5.9	1.88	Antagonism
IC_50_-IC_25_	0.275	0.011	4	3.2	0.001	*Synergism*
IC_25_-IC_75_	0.01	3.027	22.5	6.6	1.85	Antagonism
IC_25_-IC_50_	0.01	0.985	16.7	7.1	0.31	*Synergism*
IC_25_-IC_25_	0.01	0.011	39.4	2.5	0.05	*Synergism*
